# Tropomyosin Is Potential Markers for the Diagnosis and Prognosis of Bladder Cancer

**DOI:** 10.1155/2022/6936262

**Published:** 2022-06-13

**Authors:** Yunkun Yan, Jianchang Li, Mushi Ye, Zhuo Li, Sining Li

**Affiliations:** Department of Urology, Affiliated Hospital of Guangdong Medical University, Zhanjiang, Guangdong, China

## Abstract

**Objective:**

To investigate the correlation between tropomyosin (TM) and clinical characteristics of bladder cancer. In addition, the relationship between TM and immune cell infiltration in bladder cancer was further analyzed.

**Methods:**

Based on The Cancer Genome Atlas (TCGA) database, the relationship between TM expression and clinicopathological features in bladder cancer was analyzed. Receiver operating characteristic (ROC) curve was used to evaluate the value of TM as a diagnostic marker for bladder cancer. Univariate and multivariate Cox regression was used to analyze the independent factors affecting the prognosis of patients with bladder cancer. The relationship between TM and immune cell infiltration was analyzed.

**Results:**

ROC curve showed that TPM1, TPM2, and TPM3 had significant diagnostic ability (AUC was 0.845, 0.848, and 0.873, respectively). The high expression of TPM1 and TPM2 is associated with poor overall and disease-specific survival in patients with bladder cancer (*P* < 0.05). Multivariate Cox analysis showed that age and TPM1 were independent prognostic factors. The expression levels of TPM1, TPM2, TPM3, and TPM4 in low grade bladder cancer were lower than those in high grade bladder cancer (*P* < 0.05). TPM1 and TPM2 are positively correlated with the infiltration of macrophages and NK cells in bladder cancer. TPM3 is positively associated with Th2. TPM4 is positively correlated with Th1 cells, macrophages, and neutrophils (*P* < 0.05).

**Conclusions:**

TPM1 and TPM2 are effective markers for the diagnosis of bladder cancer. TPM1 is an independent prognostic factor for bladder cancer. TM is also associated with the infiltration of various immune cells in bladder cancer. TM may have influenced the development of bladder cancer through immune inhibition.

## 1. Introduction

Bladder cancer is the eleventh most common cancer in the population and the seventh most common cancer in men [1]. Muscle invasive bladder cancer (MIBC) has a high degree of malignancy. Current treatment of bladder cancer is still mainly surgical treatment. At present, targeted therapy and immunotherapy have shown great application prospects in MIBC, but there are still some problems such as low response rate and drug resistance [2].

The human genome contains four tropomyosin (TM) family genes, namely, TPM1, TPM2, TPM3, and TPM4. TM is not only present in muscle cells but also regulates cell viability and differentiation in other cells, and changes in the tropomyosin gene expression will directly affect changes in cell morphology [3]. At present, there are there few reports about the correlation between TM and bladder cancer. TPM1 has been found to be a promising diagnostic and prognostic marker for bladder cancer [4, 5]. The biological mechanism of TM in bladder cancer remains unclear. In addition, some studies have reported that the occurrence and development of bladder cancer is also related to the infiltration of immune cells [6, 7]. However, the association between TM and immune cell residence in bladder cancer has not been reported.

Therefore, we want to further analyze whether other genes in TM family are also a promising marker for bladder cancer diagnosis and prognosis as TPM1 has been reported in literatures. In addition, we want to further analyze the correlation between TM and immune cell infiltration of bladder cancer. To discuss whether TM affects the occurrence and development of bladder cancer through immunological mechanisms, this study will be conducted using appropriate bioinformatics analysis methods based on multiple databases including TCGA.

## 2. Materials and Methods

### 2.1. RNAseq Data Download and Data Analysis of Bladder Cancer

Download RNAseq data in HTSEQ-FPKM format (fragments per kilobase per million) from BLCA project in the TCGA database (https://portal.gdc.cancer.gov/). RNAseq data in FPKM format is converted into log2 data. Samples included 414 bladder cancer samples and 19 adjacent normal tissue samples. The significance of expression level was as follows: ns, *P* ≥ 0.05, ^∗^*P* < 0.05, ^∗∗^*P* < 0.01, and ^∗∗∗^*P* < 0.001. ROC analysis uses the “pROC” package (version 1.17.0.1). The area values under the ROC curve are between 0.5 and 1. The closer AUC is to 1, the better the diagnostic effect is. The abscissa is false positive rate (FPR), and the ordinate is true positive rate (TPR). “Survival” package (version 3.2-10) is used for statistical analysis of survival data, and “SurvMiner” package (version 0.4.9) is used for visualization. Univariate and multivariate Cox regression was used to analyze the independent factors affecting the prognosis of patients with bladder cancer. The prognostic data used in the analysis came from a Cell article [8].

### 2.2. Correlation Analysis of TM and Immunoinvasion in Bladder Cancer

The single-sample GSEA (SSGSEA) method in the GSVA package (1.34.0 version) of *R* (3.6.3 version) was used to analyze the infiltration of immune cells in bladder cancer. The markers and classification of 24 kinds of immune cells were obtained from an article of Immunity [9].

### 2.3. Statistical Methods

All analytical methods were performed using *R* (V.3.6.3). The visualization of the image was completed using GGplot2 (version 3.3.3) on xiantao academic platform (https://www.xiantao.love/). Wilcoxon rank sum test, chi-square test, Fisher exact test, and logistics regression were used to analyze the correlation between TM and clinical indicators of samples. Spearman correlation analysis was used to analyze the correlation between TM and immune cell infiltration level in bladder cancer. *P* value less than 0.05 was considered statistically significant.

## 3. Results

### 3.1. TM Can Be Used as an Effective Diagnostic Marker for Bladder Cancer

**Figure 1:**
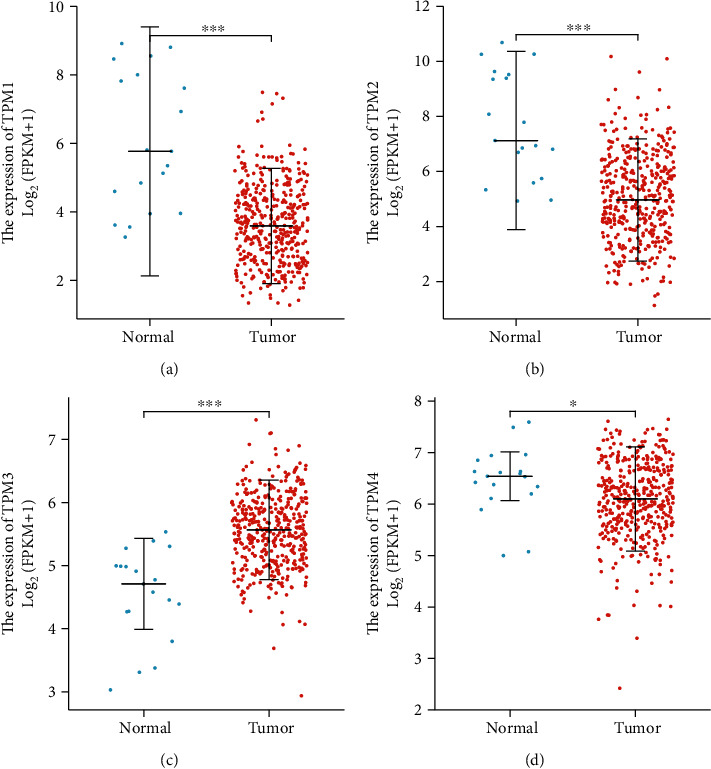
TM expression level in bladder cancer. (a) The expression of TPM1 in bladder cancer tissues and adjacent tissues. (b) The expression of TPM2 in bladder cancer tissues and adjacent tissues. (c) The expression of TPM3 in bladder cancer tissues and adjacent tissues. (d) The expression of TPM4 in bladder cancer tissues and adjacent tissues.

**Figure 2:**
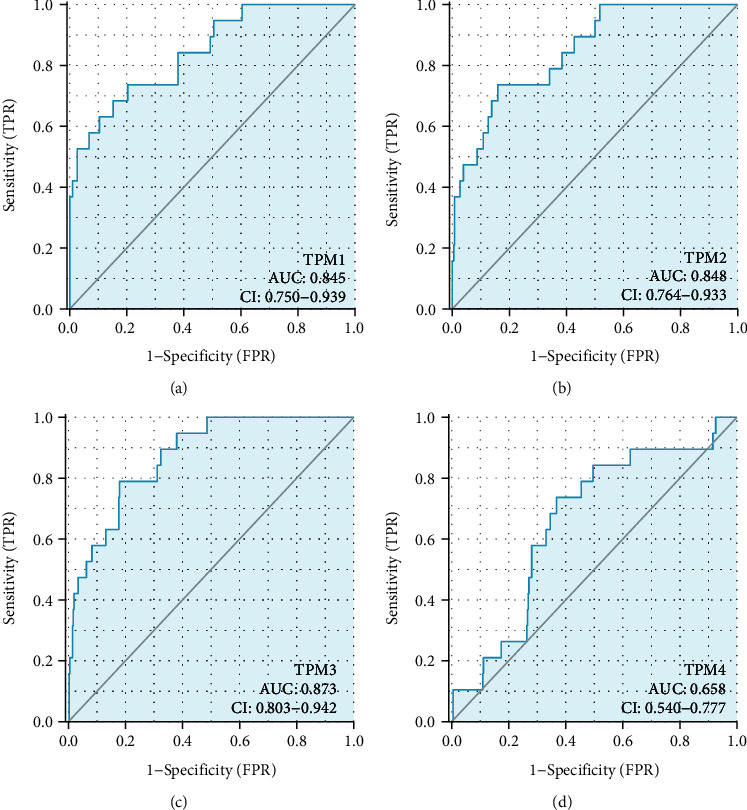
ROC curve analysis of TM prediction of bladder cancer. (a) ROC curve showed the efficacy of TPM1 expression level in distinguishing bladder cancer tissue from nontumor tissue. (b) ROC curve showed the efficacy of TPM2 expression level in distinguishing bladder cancer tissue from nontumor tissue. (c) ROC curve showed the efficacy of TPM3 expression level in distinguishing bladder cancer tissue from nontumor tissue. (d) ROC curve showed the efficacy of TPM4 expression level in distinguishing bladder cancer tissue from nontumor tissue.

We analyzed the TM expression in 414 bladder cancer specimens and 19 adjacent normal tissue specimens. The results showed that TPM1, TPM2, and TPM4 were underexpressed in bladder cancer tissues, while TPM3 was overexpressed in bladder cancer tissues (Figures 1(a)–1(d)). ROC curve was used to analyze the accuracy of TM expression level in the diagnosis of bladder cancer. ROC curve showed that TPM1, TPM2, and TPM3 had significant accuracy in the diagnosis of bladder cancer (AUC was 0.845, 0.848, and 0.873, respectively) (Figures 2(a)–2(d)). These results indicated that TPM1, TPM2, and TPM3 could be ideal biomarkers for the diagnosis of bladder cancer.

### 3.2. TM Can Be Used as Effective Markers to Predict OS and Disease-Specific Survival in Patients with Bladder Cancer

**Figure 3:**
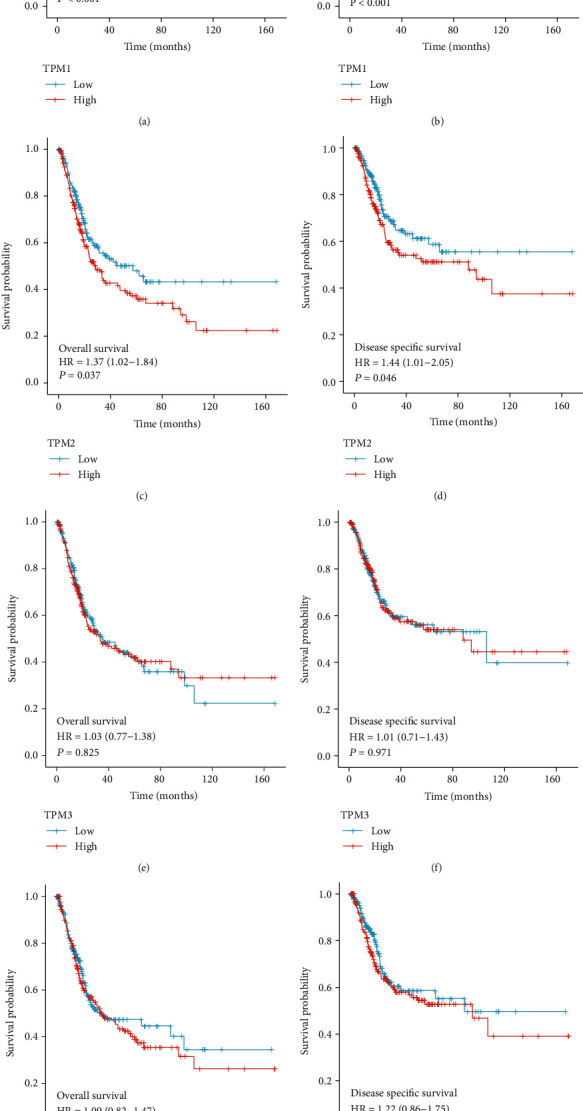
Relationship between TM expression and overall and disease-specific survival in patients with bladder cancer. (a) Relationship between TPM1 expression and overall survival in patients with bladder cancer. (b) Relationship between TPM1 expression and disease-specific survival in patients with bladder cancer. (c) Relationship between TPM2 expression and disease-specific survival in patients with bladder cancer. (d) Relationship between TPM2 expression and disease-specific survival in patients with bladder cancer. (e) Relationship between TPM3 expression and overall survival in patients with bladder cancer. (f) Relationship between TPM3 expression and disease-specific survival in patients with bladder cancer. (g) Relationship between TPM4 expression and disease-specific survival in patients with bladder cancer. (h) Relationship between TPM4 expression and disease-specific survival in patients with bladder cancer.

The high expression of TPM1 and TPM2 was associated with poor overall and disease-specific survival in bladder cancer patients (*P* < 0.05). There was no significant correlation between the TPM3 and TPM4 expression and overall survival and disease-specific survival in patients with bladder cancer (Figures 3(a)–3(h)). Univariate analysis showed that age, TPM1, and TPM2 were prognostic factors for bladder cancer (*P* < 0.05) (Table 1). Multivariate Cox analysis showed that age and TPM1 were independent prognostic factors (*P* < 0.05) (Table 1).

### 3.3. Correlation between TM and Clinical Indicators of Patients

**Figure 4:**
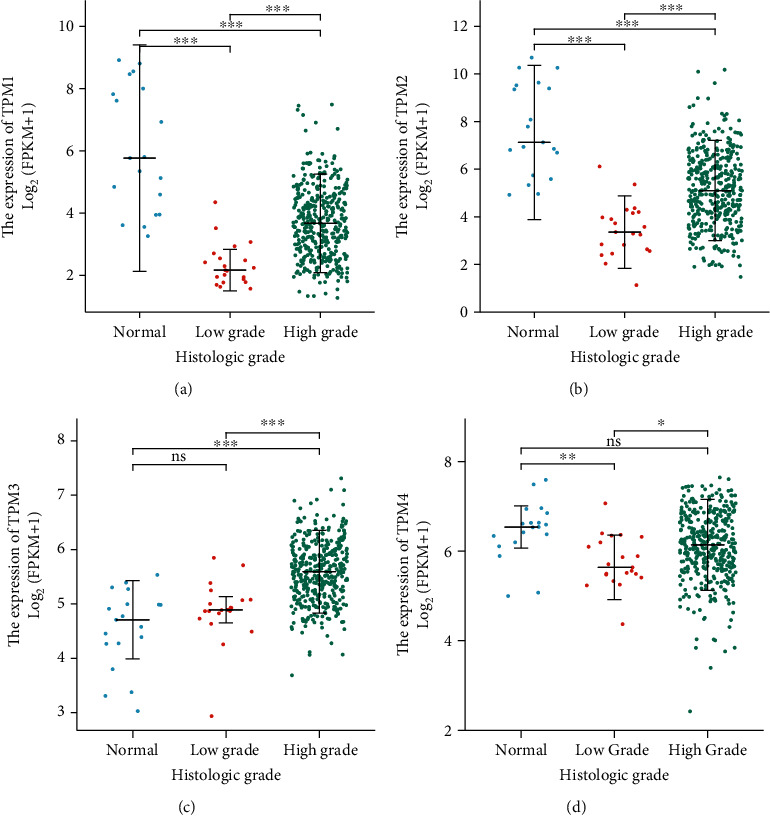
Relationship between TM expression and histological grade of bladder cancer. (a) Relationship between TPM1 expression and histological grade of bladder cancer. (b) Relationship between TPM2 expression and histological grade of bladder cancer. (c) Relationship between TPM3 expression and histological grade of bladder cancer. (d) Relationship between TPM4 expression and histological grade of bladder cancer.

We further used logistics regression to analyze the correlation between TM and clinical indicators of samples. The results showed that the expression level of TPM1 was affected by *N* stage, gender, and histologic grade (*P* <0.05) (Table 2). The expression level of TPM2 was affected by *N* stage, age and histologic grade (*P* < 0.05) (Table 3). The expression level of TPM3 is affected by histologic grade (*P* < 0.05) (Table 4). The expression level of TPM4 has not been affected by clinical indicators (Table 5). The expression levels of TPM1, TPM2, TPM3, and TPM4 in low grade bladder cancer were lower than those in high grade bladder cancer (*P* < 0.05) (Figures 4(a)–4(d)).

### 3.4. Relationship between TM Expression and Immune Cell Infiltration in Bladder Cancer

**Figure 5:**
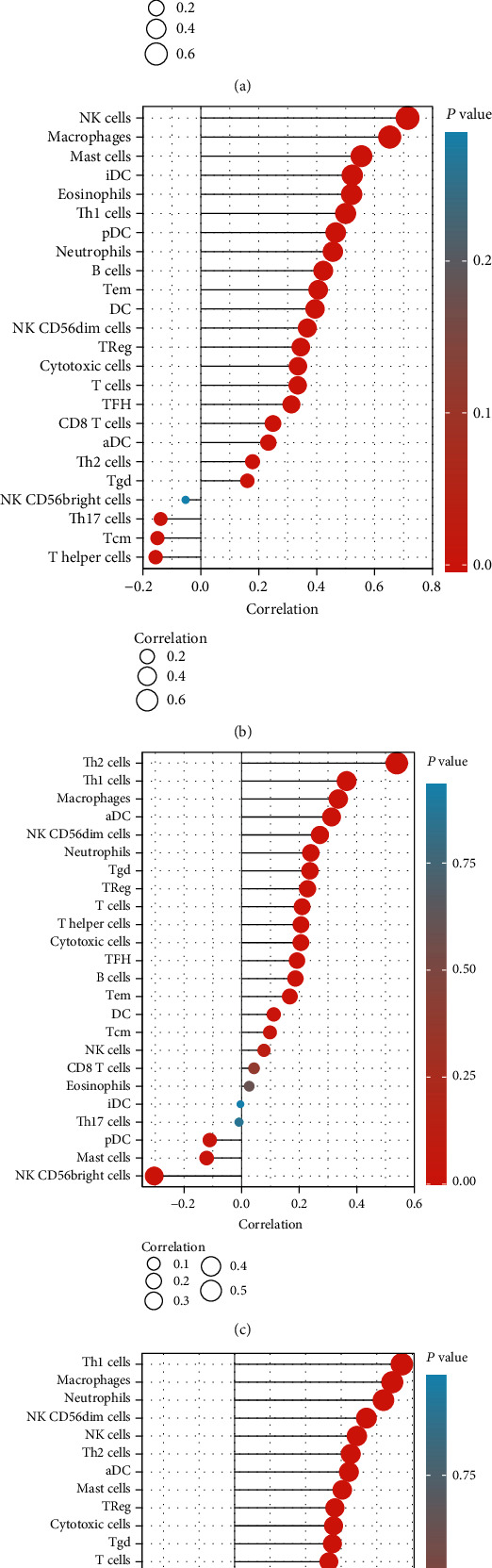
Lollipop plot shows the correlation between TM expression and immune cell infiltration in bladder cancer. (a) Correlation between TPM1 expression and immune cell infiltration in bladder cancer. (b) Correlation between TPM2 expression and immune cell infiltration in bladder cancer. (c) Correlation between TPM3 expression and immune cell infiltration in bladder cancer. (d) Correlation between TPM4 expression and immune cell infiltration in bladder cancer.

**Figure 6:**
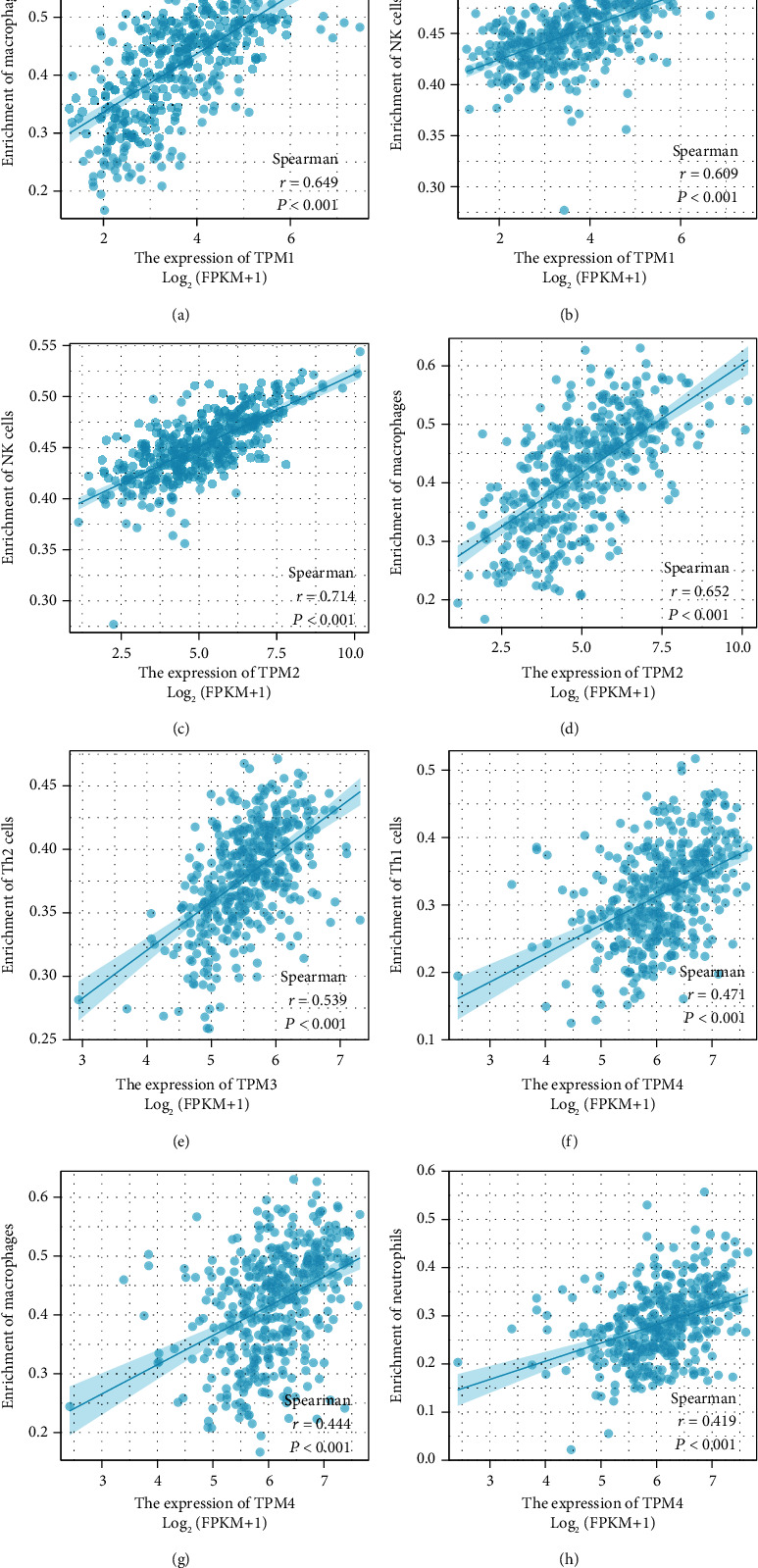
Scatter plot shows the correlation between TM expression and immune cell infiltration in bladder cancer. (a) Scatter plot shows the correlation between TPM1 expression and macrophage infiltration in bladder cancer. (b) Scatter plot shows the correlation between TPM1 expression and NK cell infiltration in bladder cancer. (c) Scatter plot shows the correlation between TPM2 expression and NK cell infiltration in bladder cancer. (d) Scatter plot shows the correlation between TPM2 expression and macrophage infiltration in bladder cancer. (e) Scatter plot shows the correlation between TPM3 expression and Th2 invasion in bladder cancer. (f) Scatter plot shows the correlation between TPM4 expression and Th1 invasion in bladder cancer. (g) Scatter plot shows the correlation between TPM4 expression and macrophage infiltration in bladder cancer. (h) Scatter plots show the correlation between TPM4 expression and neutrophil infiltration in bladder cancer.

SSGSEA was used to further analyze the relationship between TM expression and immune cell invasion in bladder cancer. The results showed that TPM1 and TPM2 were positively correlated with macrophage and NK cell infiltration in bladder cancer. TPM3 was positively correlated with Th2. TPM4 was positively correlated with Th1 cells, macrophages, and neutrophils (*P* < 0.05) (Figures 5(a)–5(d) and 6(a)–6(h)).

## 4. Discussion

Tropomyosin, a member of the actin binding protein family, was originally thought to be a myofibrillary structural protein involved in the contractile activity of skeletal and cardiac muscles [10]. Although TM has been studied for a long time, further studies have shown that TM is not only present in skeletal muscle and cardiac muscle cells but also expressed in almost all cells [3]. The TM family genes consists of four genes: TPM1, TPM2, TPM3, and TPM4. At present, most of the researches on TM family genes only focus on one or two tropomyosin genes and lack of systematic studies. Studies have shown that TPM1 is a very important tumor suppressor, and its expression level is low in many solid tumors [11]. At present, there are few reports about the correlation between TM and bladder cancer, especially the correlation between TM and immune cell infiltration of bladder cancer remains unclear. Therefore, based on the TCGA database, bioinformatics analysis method was adopted to comprehensively analyze the correlation between TM and bladder cancer. The great potential of TM in diagnosing and predicting bladder cancer is expected to be explored. In addition, this study also analyzed the correlation between TM and the presence of immune cells in bladder cancer, hoping to provide more research evidence for the further study of the immunological mechanism of bladder cancer.

Our study showed that TPM1, TPM2, and TPM4 were underexpressed in bladder cancer tissues. The above results are consistent with current literature reports [12, 13]. It has been reported that TPM1 and TPM2 are highly expressed in normal urothelial tissues, but the expression of TPM1 and TPM2 is decreased in the early stage of bladder cancer, which may be a marker event of the occurrence and development of bladder cancer [12]. Cell experiments have confirmed that TPM1 inhibits the proliferation of bladder cancer cells and promotes cell apoptosis [5]. Therefore, the low expression of TPM1, TPM2, and TPM4 in the tumor tissues of bladder cancer may be beneficial to the rapid proliferation of bladder cancer cells, indicating the development of bladder cancer. In addition, ROC curve indicated that TPM1, TPM2, and TPM3 had significant accuracy in the diagnosis of bladder cancer. Therefore, TPM1, TPM2, and TPM3 are expected to be new markers for the diagnosis of bladder cancer and will play a potentially great value in clinical conversion application.

We further explored the application value of TM in predicting the prognosis of bladder cancer. The high expression of TPM1 and TPM2 is associated with poor overall and disease-specific survival in bladder cancer patients. Multivariate Cox analysis showed that age and TPM1 were independent prognostic factors. In conclusion, TPM1 can be used as an effective marker to predict the survival and prognosis of bladder cancer patients.

When we analyzed the correlation between TM and clinical indicators of bladder cancer, we found that the expression of TM was affected by the histological grade of bladder cancer. The expression levels of TPM1, TPM2, TPM3, and TPM4 in low-grade bladder cancer were lower than those in high-grade bladder cancer. This may explain why the high expression of TM has a worse prognosis when we discuss the relationship between TM and prognosis.

A current study of bladder cancer has shown that muscle-invasive bladder cancer has a different pattern of immune cell infiltration than normal tissue, and a model based on the difference in immune cell infiltration may offer promising predictive value [7]. Current studies have suggested that the presence of CD8+ T cells in bladder cancer is a favorable prognostic factor, while the increased expression of PD-L1 and the presence of tumor-related macrophages are a negative prognostic factor [14]. In this study, we focused on whether the TM play a role in the infiltration of immune cells in bladder cancer. We found that TPM1 and TPM2 were positively correlated with macrophage and NK cell infiltration in bladder cancer. TPM3 was positively correlated with Th2. TPM4 was positively correlated with Th1 cells, macrophages, and neutrophils. We have previously observed that TPM1, TPM2, and TPM4 are low expressed in bladder cancer, and therefore, the infiltration of NK cell, macrophages, neutrophils, and Th1 may be reduced accordingly in bladder cancer. Therefore, we infer that the low expression of TPM1, TPM2, and TPM4 is a mechanism of immunosuppression in bladder cancer. Therefore, TM is expected to be used as a predictor of immune cell infiltration in bladder cancer and a potential therapeutic target.

In conclusion, TPM1 and TPM2 are effective markers for the diagnosis of bladder cancer. TPM1 is an independent prognostic factor for bladder cancer. TM is also associated with the infiltration of various immune cells in bladder cancer. TM may have influenced the development of bladder cancer through immune inhibition.

## Figures and Tables

**Table 1:** Univariable and multivariable analysis of OS in patients with bladder cancer.

Characteristics	Total (*N*)	Univariate analysis		Multivariate analysis	
		Hazard ratio (95% CI)	*P* value	Hazard ratio (95% CI)	*P* value
Age	413				
< =70	233	Reference			
>70	180	1.421 (1.063-1.901)	0.018	1.469 (1.096-1.970)	0.010
Gender	413				
Female	109	Reference			
Male	304	0.849 (0.616-1.169)	0.316		
Pathologic stage	411				
Stage I	4	Reference			
Stage II, stage III, and stage IV	407	9132093.375 (0.000-Inf)	0.993		
TPM1	413				
Low	207	Reference			
High	206	1.718 (1.273-2.318)	<0.001	1.807 (1.254-2.602)	0.001
TPM2	413				
Low	207	Reference			
High	206	1.368 (1.019-1.836)	0.037	0.947 (0.661-1.355)	0.764
TPM3	413				
Low	207	Reference			
High	206	1.033 (0.772-1.382)	0.825		
TPM4	413				
Low	207	Reference			
High	206	1.094 (0.816-1.468)	0.547		

**Table 2:** Logistics regression of single gene TPM1.

Characteristics	Total (*N*)	Odds ratio (OR)	*P* value
T stage (T2, T3, and T4 vs. T1)	380	4.287 (0.627-84.290)	0.195
*N* stage (N1, N2, and N3 vs. N0)	370	2.611 (1.683-4.094)	<0.001
M stage (M1 vs. M0)	213	2.901 (0.848-11.381)	0.098
Pathologic stage (stage II, stage III, and stage IV vs. stage I)	412	3.059 (0.388-62.130)	0.335
Gender (male vs. female)	414	0.479 (0.304-0.748)	0.001
Age (>70 vs. <=70)	414	1.040 (0.705-1.535)	0.843
Histologic grade (high grade vs. low grade)	411	22.162 (4.552-399.669)	0.003

**Table 3:** Logistics regression of single gene TPM2.

Characteristics	Total (*N*)	Odds ratio (OR)	*P* value
T stage (T2, T3, and T4 vs. T1)	380	4.523 (0.661-88.920)	0.179
*N* stage (N1, N2, and N3 vs. N0)	370	2.204 (1.425-3.439)	<0.001
M stage (M1 vs. M0)	213	3.092 (0.903-12.136)	0.079
Pathologic stage (stage II, stage III, and stage IV vs. stage I)	412	46226230.510 (0.000-NA)	0.993
Gender (male vs. female)	414	0.723 (0.464-1.120)	0.148
Age (>70 vs. <=70)	414	1.484 (1.005-2.196)	0.048
Histologic grade (high grade vs. low grade)	411	10.419 (2.972-65.963)	0.002

**Table 4:** Logistics regression of single gene TPM3.

Characteristics	Total (*N*)	Odds ratio (OR)	*P* value
T stage (T2, T3, and T4 vs. T1)	380	3.979 (0.582-78.223)	0.219
*N* stage (N1, N2, and N3 vs. N0)	370	1.262 (0.824-1.937)	0.285
M stage (M1 vs. M0)	213	0.657 (0.168-2.243)	0.512
Pathologic stage (stage II, stage III, and stage IV vs. stage I)	412	45997389.201 (0.000-NA)	0.993
Gender (male vs. female)	414	0.975 (0.629-1.512)	0.911
Age (>70 vs. <=70)	414	1.000 (0.678-1.475)	1.000
Histologic grade (high grade vs. low grade)	411	10.313 (2.941-65.289)	0.002

**Table 5:** Logistics regression of single gene TPM4.

Characteristics	Total (*N*)	Odds ratio (OR)	*P* value
T stage (T2, T3, and T4 vs. T1)	380	65249103.465 (0.000-NA)	0.993
*N* stage (N1, N2, and N3 vs. N0)	370	1.157 (0.755-1.776)	0.503
M stage (M1 vs. M0)	213	1.058 (0.296-3.623)	0.928
Pathologic stage (stage II, stage III, and stage IV vs. stage I)	412	46226232.235 (0.000-NA)	0.993
Gender (male vs. female)	414	0.760 (0.488-1.177)	0.220
Age (>70 vs. <=70)	414	1.000 (0.678-1.475)	1.000
Histologic grade (high grade vs. low grade)	411	2.084 (0.848-5.601)	0.121

## Data Availability

We did not create any data, and all the results we analyzed in our paper were based data from public database. So, we do not have any data that could be uploaded to a repository.
